# Structural Insights into Membrane Fusion Mediated by Convergent Small Fusogens

**DOI:** 10.3390/cells10010160

**Published:** 2021-01-15

**Authors:** Yiming Yang, Nandini Nagarajan Margam

**Affiliations:** Department of Microbiology and Immunology, Dalhousie University, Halifax, NS B3H 4R2, Canada; nn760994@dal.ca

**Keywords:** fusogen, SNARE, FAST, atlastin, spanin, myomaker, myomerger, membrane fusion

## Abstract

From lifeless viral particles to complex multicellular organisms, membrane fusion is inarguably the important fundamental biological phenomena. Sitting at the heart of membrane fusion are protein mediators known as fusogens. Despite the extensive functional and structural characterization of these proteins in recent years, scientists are still grappling with the fundamental mechanisms underlying membrane fusion. From an evolutionary perspective, fusogens follow divergent evolutionary principles in that they are functionally independent and do not share any sequence identity; however, they possess structural similarity, raising the possibility that membrane fusion is mediated by essential motifs ubiquitous to all. In this review, we particularly emphasize structural characteristics of small-molecular-weight fusogens in the hope of uncovering the most fundamental aspects mediating membrane–membrane interactions. By identifying and elucidating fusion-dependent functional domains, this review paves the way for future research exploring novel fusogens in health and disease.

## 1. Introduction

Membrane fusion is a universal reaction that mediates a myriad of biological events, such as fertilization, organ and tissue growth, cancer metastasis, and multi-nucleated giant cell formation during an immune response [1,2,3,4]. Besides facilitating these phenomena on a molecular scale, membrane fusion plays an indispensable role in the secretion of biomolecules, trafficking of proteins, and the homeostatic maintenance of the endoplasmic reticulum and mitochondrial networks [5,6]. Despite the diversity in the organisms and cell types that utilize cell fusion in their normal physiology and pathology, the fusion reactions share common features. All the fusion processes can be divided into steps: aggregation of the membranes, lipid bilayers immediate contact, rearrangement of outer lipids resulting in the formation of a stalk, stalk expansion yielding the hemifusion diaphragm, fusion pore formation, and pore expansion [7]. Facilitating these sequential events are a broad array of fusogens. Due to the large difference of the fusing membranes, some fusogens mediate fusion by presenting on only one of the fusing membranes, termed unilateral mechanism, while others present on both membranes, termed bilateral mechanism. In addition, fusion events are classified into homotypic or heterotypic; in a homotypic fusion, it is mediated by the interaction of the same type of protein (e.g., Influenza HA2), and in heterotypic fusion, it is mediated by different proteins (e.g., N-ethylmaleimide-sensitive factor attachment protein receptor (SNARE) complex and myomaker-myomerger). To initiate membrane fusion, the fusogens have evolved to contain specific regions, such as hydrophobic motifs and residues, that aid in membrane curvature, lipid rearrangement and mixing, and pore formation.

The first fusogens identified were the viral fusogens encoded by enveloped viruses, which play a vital role in merging membranes to mediate viral entry into target cells. A detailed description on the structure of the different classes of enveloped viral fusogens and the mechanism by which they mediate virus–cell fusion are explained in other reviews [8,9,10]. Briefly, enveloped viral fusogens are divided into three types based on structural discrepancies: (1) class I viral fusogens prominently contain α-helixes, with their fusion peptides at or near the N-terminus; (2) class II viral fusogens differ from class I fusogens by the primary presence of β-sheets structure with internal fusion peptides formed as loops at the tips of β-strands; and (3) class III viral fusogens are comprised of both α-helices and β-sheets along with an internal hydrophobic fusion loop for lipid interaction [8,10]. Enveloped viral fusogens mediate heterotypic interaction (viral and cell membrane) and are present unilaterally.

Another set of well-studied fusogens are the cell–cell fusion proteins, including syncytin, EFF-1, and HAP-2 that primarily mediate developmental fusion. The syncytin is an endogenous retroviral envelope protein required for cytotrophoblast fusion during placenta development [11]. The N- and C-terminal heptad repeats region of syncytin shared 44% and 62% sequence identity with the corresponding N-peptide and C-peptide of HIV gp160, respectively, indicating the similar structural profiling between the syncytin and the class I viral fusogens [12]. Besides analogous structure, syncytins present unilaterally, just like class I viral fusogens, and induce fusion of placental cytotrophoblast in vivo [11]. On the other hand, the somatic fusogen EFF-1 and the sexual gamete fusogen HAP2 resemble class II viral fusogens, containing three β-sheet-rich domains [13,14,15,16]. EFF-1 mediate homotypic or heterotypic bilateral membrane fusion, while the HAP2 mediates fusion bilaterally or unilaterally depending on the organism [17,18,19,20,21]. Given that the three cell–cell fusogens possess structural similarities with viral fusogens, we deduce that these fusogens might have descended from a common ancestor and evolved into divergent fusogens acting to achieve distinct functional outcomes (heterotypic or homotypic fusion) with discrepant modalities of actions (unilateral or bilateral).

Besides evolving from a common progenitor, certain small fusogens may have evolved independently. They often possess molecular weights varying from 10–30 kDa. Examples of these small fusogens are soluble SNARE proteins, atlastins, spanins, myomaker, myomerger, and fusion-associated small transmembrane (FAST) proteins. Due to their compact structural simplicity, these small fusogens potentially harbor the most fundamental units that play a role in membrane curvature, lipid interaction, and pore formation/expansion. Therefore, these small fusogens serve as critical models to study the mystery of membrane fusion. This review will highlight both similarities and differences in the structures and activities of these small fusogens, interrogating their various functional domains and motifs in the establishment of membrane fusion and comparing and contrasting molecular features of the various members within this group. Collectively, we hope to pave the way for probing a comprehensive understanding of the basic molecular components conferring fusogenic activity.

## 2. Intravesicular Fusogen: SNARE Family

**Figure 1:**
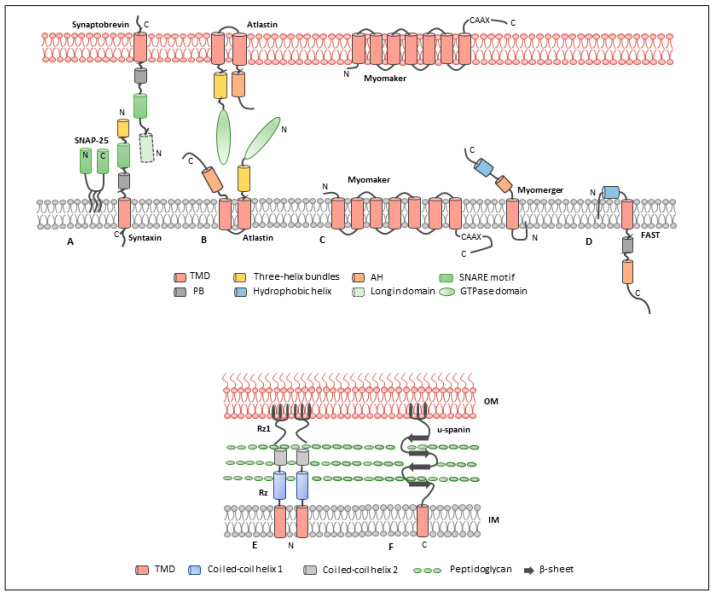
Hallmark features of fusion proteins. (**A**) Structures of N-ethylmaleimide-sensitive factor attachment protein receptor (SNARE) proteins: SNAP-25 contains two SNARE motifs connected by a linker that is palmitoylated; syntaxin consists of an N-terminal three-helix bundle, followed by a SNARE motif and a TMD linked by a polybasic region; Synaptobrevin involves either a short peptide or a longin domain at N-terminal (domain highlighted with dashed line); (**B**) atlastin is composed of a cytosolic GTPase domain at the N-terminus, followed by three helical bundles, two closely spaced transmembrane domains, and a cytosolic amphipathic helix at C-terminus; (**C**) myomaker contains seven transmembrane domains, extending the N-terminus to the extracellular space and locating the C-terminus in the cytoplasm. Myomerger is composed of a short hydrophobic N-terminal peptide and a single transmembrane domain, followed by an amphipathic helix and an adjacent hydrophobic helix at C-terminus; (**D**) the fusion-associated small transmembrane (FAST) protein possesses a hydrophobic helix in the N-terminal ectodomain, a TMD, and a C-terminal endodomain comprising a polybasic motif and an amphipathic α-helix; (**E**) two-component spanins—Rz contains an N-terminal transmembrane domain in the inner membrane (IM) and two coiled-coil helices at the C-terminus, while Rz1 comprises a lipid anchor at the N-terminus inserted in the outer membrane (OM) and a proline-rich motif (PPAPP) in the central region; and (**F**) u-spanin utilizes N-terminal fatty acyl chains inserted in the OM, followed by a periplasmic domain composed of β-stands and a C-terminal TMD in the IM.

The SNARE complex is comprised of three proteins—synaptosomal-associated protein-25 (SNAP-25), syntaxin, and synaptobrevin (vesicle-associated membrane protein, VAMP). The molecular weight of the SNARE protein superfamily members ranges from ~20 kDa to 30 kDa, with approximately 60 members present in yeasts and mammals [22]. Syntaxin and SNAP-25 are t-SNAREs contributed by the target membrane, while synaptobrevin is a v-SNARE from the vesicular membrane. All SNARE proteins contain a common SNARE motif and structurally divergent N- and C-termini (Figure 1A). Specifically, syntaxin is composed of an N-terminal domain, a conserved SNARE motif, and inserted into the membrane via a transmembrane domain (TMD) at the C-terminus. Synaptobrevin contains either a longin domain or a short unstructured peptide at N-terminus, followed by the SNARE motif and a transmembrane domain anchored on the membrane. SNAP-25 family proteins lack a transmembrane domain: two SNARE motifs are linked by a linker region and then inserted into the membrane by palmitoylation modification [23,24,25]. SNAREs appear to lie at the center of the membrane fusion mediating vesicle fusion within the endomembrane system and the vesicle exocytosis, which includes the ER, the Golgi, endosomes, and lysosomes [26,27]. SNAREs mediate fusion through the *trans*-SNARE complex that, “zipping” from the distal N-terminal region to the proximal C-terminal region, brings the two opposing membranes closer and eventually completing the fusion of the membranes [28,29,30].

### 2.1. SNARE Complex and SNARE Motifs

In a typical SNARE complex, syntaxin and synaptobrevin each contribute one SNARE motif, whereas SNAP-25 contributes two [23,24,25]. The SNARE motif is a conserved component of SNARE proteins and is amphipathic in nature. Structurally, the SNARE motif is made up of heptapeptide repeats that adopt α-helices, with all the hydrophobic residues on one side of the helix and hydrophilic residues on the other [31]. SNARE motifs are unstructured in their monomeric form. But on the membrane contact, these proteins assemble into a four-helical *trans*-complex termed “core complex” when they interact with one another [32,33,34]. Of note, a hydrophilic layer exists in the middle of the bundle consisting of three glutamine residues—one contributed by syntaxin and two by SNAP-25, and one arginine residue contributed by synaptobrevin [25]. This layer is important for stabilizing the core SNARE complex. This *trans*-complex proceeds from a loose state (only the SNARE motifs are “zipped up”) to a tight state (the zippering process is mostly completed), generating enough energy to overcome the energy barrier between two opposite phospholipid and mediating membrane fusion. During fusion, the strained *trans*-complex relaxes into a *cis*-configuration. *Cis*-complexes are disassembled by the N-ethylmaleimide-sensitive factor (NSF) together with SNAPs (soluble NSF attachment proteins). The SNARE proteins are then separated by protein sorting, i.e., endocytosis [35].

### 2.2. N-Terminal Domains

Diverse N-terminal regions have critical functions in regulating SNARE-mediated fusion cascade. For instance, the N-terminal of syntaxin consists of two elements, a short unstructured peptide followed by a three-helical (Habc) domain, that execute distinct biological functions. The short peptide provides a binding site for Munc18-1, which is required for synaptic vesicle fusion, while the Habc domain stabilizes this binding and maintains the normal level of Munc18-1, like syntaxin-1 [36]. In addition, the Habc domain binds to the SNARE motif in an autoinhibited manner, rendering the protein in a closed conformation, thus inhibiting syntaxin function [37]. Furthermore, this domain regulates the localization of syntaxin to the cell surface [38]. Unlike syntaxins, however, synaptobrevins contain either a short peptide (e.g., brevinin) or a long domain of 120–140 aa termed longin domain (e.g., VAMP7, Ykt6, Sec22). The longin domain functions to either inhibit the assembly of *trans*-SNARE complex or interact with other proteins to mediate intracellular trafficking [23,39,40,41]. In the PC12 cell model of growing neurites, deletion of the longin domain of VAMP7 activates SNARE complex assembly, supporting an autoinhibitory function of the longin domain [42]. As to SNAP-25, they present N-terminal extensions. For instance, the N-terminal of SNAP-25 family member, Vam7, contains a PX domain, functioning as a phosphatidylinositol phosphate-binding motif that provides a phosphatidylinositol 3-phosphate (PI3P)-specific membrane anchor [43,44] and promotes *trans*-SNARE complex assembly [45]. Collectively, the N-terminal domain in syntaxins and synaptobrevins acts to either inhibit SNARE complex assembly or enable proper trafficking and localization for a functional SNARE protein, whereas in the SNAP-25 family, the N-terminal domain promotes SNARE complex assembly.

### 2.3. Linker Region

Found in syntaxin and synaptobrevin, the juxtamembrane linker region connecting the SNARE motif and the transmembrane domain is a polybasic region required for efficient membrane fusion [46,47]. In vitro and in vivo studies analyzing this linker region of syntaxin have shown that any change in the length of the helix or amino acid residues significantly reduces fusion efficiency, suggesting that the length and the property of the helix are important to mediate membrane fusion [46,48]. In addition, the linker region of VAMPs contains highly conserved and positively charged tryptophan and lysine residues. These residues have been shown to interact with membrane phospholipids by bridging the negatively charged membrane leaflets. Hydrophobic tryptophan residues determined the immersion depth of the protein on the membrane that is thought to be involved in the fusion reaction [49,50]. Moreover, both basic and hydrophobic residues are required by the VAMP2 linker region for promoting membrane fusion by destabilizing the lipid bilayer [51]. Furthermore, lipid mixing analysis applied on synaptobrevin confirmed that this juxtamembrane region is required for stalk and pore formation during fusion [52]. As a result, the length and amino acid fidelity of the linker region are critical factors for SNAREs-mediating membrane fusion.

### 2.4. C-Terminal Transmembrane Domain

Syntaxin and synaptobrevin are anchored to the membranes by a transmembrane domain (TMD) at the C-terminus. The TMD consists of both α-helixes and β-sheets, and this conformation is required for the flexibility and stability of the domain to perform its function. Deletion or shortening of the TMD leads to fusion defects, underscoring the importance of the TMD in facilitating membrane pore formation or pore expansion [53,54]. Furthermore, the retention of amino acid specificity and structural stability in TMD is of paramount importance in enabling vesicular fusion. For example, mutation of synaptobrevin TMD with poly-leucine significantly impaired fusion of vesicles, whereas replacement with other β-branched residues, such as isoleucine or valine, retained the function [55,56]. As preluded in the introduction, membrane fusion is mediated by the formation of hemifusion stalk followed by pore expansion to mix the internal contents. The TMD of SNARE proteins plays a major role in hemifusion to fusion transition evidenced in that the deletion mutant of v-SNARE Snc2p TMD arrests the fusion process at hemifusion [57]. Additionally, replacing uncharged residues in the C-terminus of synaptobrevin TMD with charged residues inhibits pore expansion, suggesting that the movement of uncharged residues into the hemifusion stalk is necessary for pore expansion [54,58].

## 3. ER-Shaping Protein: Atlastin

In addition to intracellular vesicle fusion mediated by SNAREs, atlastin (ATL) is an ER-shaping protein that belongs to the dynamin superfamily of GTPases primarily responsible for generating and maintaining the unique reticular morphology of the ER [59]. Knockdown or overexpression of dominant-negative form of atlastin results in the deformation of Golgi and ER morphology and disrupts tubular connection [60,61]. In addition, mutations of atlastin dramatically reduced neuronal ER tubules in dendrites of *Caenorhabditis elegans* sensory neuron, PVD. This speaks to the requirement of atlastin to mediate ER fusion and proper organization of the ER network [62]. Atlastin contains a cytosolic GTPase domain at the N-terminus, followed by a three-helix bundle (3HB, middle domain), two closely spaced transmembrane domains, and a cytosolic amphipathic helix at the C-terminus [63] (Figure 1B). Atlastins distribute on both ER membranes, mediating membranes fusion that beginning with dimerization triggered by GTP binding. When GTP is hydrolyzed by the dimer, the GTPase domain and the middle domain undergo conformational changes that bring the two membranes together.

### 3.1. GTPase Domain and Middle Domain

The GTPase domain is the most conserved region among the GTPase family members. Specific arginine residues embedded within the GTPase domain serve as crucial determinants modulating atlastin function and subsequent membrane fusion. Byrnes et al. showed that the arginine at position 77 (R77) is required for faster hydrolysis of GTP [64]. Moreover, mutation of Drosophila atlastin arginine at position 48 (R48), which is equivalent to R77 in humans, deprives atlastin of its ability to dimerize and undergo GTP binding [65]. Thus, the presence of arginine residues in the GTPase domain is vital to ATL mediating fusion.

The middle domain is comprised of a three-helix bundle that connects the GTPase domain to the transmembrane domain. The middle domain is required to stabilize atlastin dimerization, the process of which hydrolyzes GTP and generating the energy required for membrane destabilization and merging of lipid bilayer [66]. Mutation of core hydrophobic residues within the helix bundle significantly reduces GTPase activity and the resultant ER membrane fusion, suggesting that hydrophobic residues in the middle domain are indispensable for atlastin dimerization and membrane tethering [67].

### 3.2. Transmembrane Domain

Atlastins have two transmembrane domains separated by a loop of five amino acids [66,68,69]. The hydrophobic TMDs of atlastins are similar in sequence across many species, and they are functionally conserved [70]. They do not serve as mere membrane anchors; they also drive fusion. The study performed by Liu et al. demonstrated that the swapping of the TMDs with Sec61β (tail-anchored protein) or Sac1p (integral ER membrane protein) resulted in a fusion-defective construct, which reveals the vital role of TMD in the atlastin-mediated membrane fusion [71]. In addition, the distance between the two transmembrane segments is important because the insertion of seven residues into the intervening loop renders the atlastin inactive, even though membrane integration is not affected. Further, insertion of additional residues in between the TMD and the middle domain inhibits fusion as well, indicating that the specific length of the linker connecting TMD and the middle domain is required to mediate membrane curvature and fusion [67]. Thus, the specific structures of TMDs and the length of the membrane-proximal external region are essential for atlastin-mediating membrane fusion.

### 3.3. C-Terminal Amphipathic Helix

The C-terminal tail contains an amphipathic helix (AH) that is important for atlastin function. This 25-amino-acid helix lies proximal to lipid membranes and is conserved in atlastins across various species, not just limited to humans and *Drosophila* but also seen in plants [65,66,72,73]. Obviously, the shared presence of AH in a variety of species testifies its importance in guiding membrane fusion. During fusion, this region becomes more helical upon interaction with the lipid bilayer to destabilize the membranes and utilizes the energy generated by GTP hydrolysis to drives membrane fusion [66,71]. Interestingly, the whole C-terminus deletion did not dramatically decrease the GTP hydrolysis; however, this deletion blocked atlastin mediated membrane fusion. This small reduction in the GTPase activity for the C-terminal deletion may reflect the gentle effect of the C-terminal tail on atlastin oligomerization. Moreover, the deletion mutant retaining the AH domain restored ∼70% of WT fusion activity, confirming the C-terminal AH domain of atlastin is critical for membrane fusion [66].

## 4. Bacteriophage Fusogen: Spanins

Membrane fusion is integral to both the eukaryotic system and prokaryotic lives. A well-summarized review describes the mechanisms by which phages lysis the host bacteria and the pathways of the disruption of the cell membranes [74]. The phages release is always a regulated process that starting with a temporally controlled permeabilization of the cytoplasmic (inner) membrane followed by enzymatic degradation of the peptidoglycan. After the new phage particles were assembled, (1) holin triggers, resulting in micron-scale holes that release the endolysin into the periplasm (λ-like lysis pathway); or (2) the triggered pinholin causes small heptameric pinholes releasing the signal-arrest-release (SAR) endolysin from the inner membrane into the periplasm (pinholin-SAR endolysin lysis pathway). Both pathways induce peptidoglycan degradation. The degradation of peptidoglycan results in spanin activation, mediating efficient inner and outer membranes fusion, which removes the topological barrier of the outer membrane releasing phages or cytoplasmic content.

Spanins are specific proteins encoded by phages of Gram-negative bacteria that span the periplasm connecting the inner and outer membranes of the bacterial wall. Bacteria infected with spanin-deleted bacteriophages did not rupture but changed their morphology from rod-shaped to spherical shape, instead. This indicates that the spanin mediates fusion after the perforation of the inner membrane [75]. The spanin complex consists of two components—Rz (i-spanin) and Rz1 (o-spanin). They are both required for a functional spanins complex to merge the lipid membranes [76,77]. Following cleavage of the peptidoglycan layer by endolysins, the Rz in the inner membrane (IM) and Rz1 in the outer membrane (OM) oligomerize to pull the membranes and destabilize the lipid membrane to form a pore for releasing the bacteriophages [78]. Alternatively, the bacteriophage T1 encodes a single spanin termed u-spanin (gp11) that spans the inner and outer membrane and folds back to fuse the membranes to release the phages [75]. Although spanins achieve fusion of bacteria membranes, they share similar structures with cellular fusion proteins.

### 4.1. Rz

The Rz is encoded by the *Rz* gene of bacteriophages. Structurally, it inserts in a N_in_/C_out_ manner with its N-terminus comprising of the transmembrane domain in the inner membrane as well as a C-terminal periplasmic domain containing two α-helices (Figure 1E). The TMD of Rz is only required for membrane anchor because the replacement of the native TMD with the TMD of FtsI, a type II membrane protein involved in cell division, does not disrupt the function of the protein [76]. Rz, as well as Rz1 discussed below, dimerize via disulfide bonds formed between cysteine residues, and deficiency of such disulfide bonds formation results in defective lysis [79]. So, the fidelity of C-terminal cysteine residues C99 and C152 is crucial to permit protein association such that mutations of these cysteine residues obstructed Rz homodimerization [77]. In addition, the mutational analysis revealed that the hydrophobic residues at the first coiled-coil region (CC1) are important for dimerization and that the CC2 is important for interaction with Rz1 to form the spanin complex. Furthermore, the linker region that connects CC1 and CC2 is predicted to be a β-strand that could function to bring the two coiled regions together, thereby pulling the membranes close contact [77].

### 4.2. Rz1

The genetic segment encoding the 60-aa Rz1 lipoprotein is co-embedded in the reading frame with the *Rz* gene entirely. This in-frame-gene transcription could have been acquired during evolution, as phages infecting Gram-negative bacteria could have introduced both Rz and Rz1 [80]. The Rz1 is attached to the inner leaflet of the outer membrane via three fatty acyl chains and has a periplasmic domain predicted to be unstructured (Figure 1E). As mentioned, Rz1s accumulate as homodimers covalently linked by intermolecular disulfide bonds between cysteine residues C29. The Rz1 protein itself is highly unstructured but adopts a helical conformation upon interacting with the dimerized Rz [77]. The central region of the periplasmic domain comprises five proline residues, termed a proline-rich motif (PPAPP), and is important for IM–OM fusion. Also, point mutation of this region results in defective host lysis [80]. Furthermore, the leucine residue at position 50 (L50) mutating to proline causes fusion defects due to the aberrant helical formation of Rz1, suggesting the importance of helix formation during membrane fusion. These studies reveal that the presence of hydrophobic residues and helical structural regions are required for lipid binding and possibly membrane curvature to mediate fusion.

### 4.3. Unimolecular Spanin

Found in 13 families of bacteriophages, the unimolecular spanin (u-spanin) can mediate IM–OM fusion on its own instead of requiring both Rz and Rz1 to function. Structurally, the N-terminal three fatty acyl chains insert in the outer membrane, whereas the C-terminal TMD is embedded in the inner membrane. The periplasmic region in between the N- and C-termini is predominantly made up of β-strands that helps bring the inner and outer membranes in close opposition [81] (Figure 1F). Unlike TMDs of some fusogens that participate in the fusion, the TMD of u-spanin is required only for membrane anchor and not for oligomerization or membrane curvature. Besides, different from Rz and Rz1, the u-spanins do not contain cysteine residues in the periplasmic region to form disulfide bonds; however, the protein must also oligomerize to force the membranes together. In addition, comparing with the two-component spanins, no hydrophobic residues or amphipathic regions have been identified in the u-spanin. The differences between the u-spanin and the two-component spanins suggest the fusion pathways may be dramatically different yet functionally equivalent. Studies thus far have only illustrated the role of u-spanins in mediating bacteriophage release, but the underlying mechanisms by which they mediate fusion are still lacking.

## 5. Muscle Fusogens

All the aforementioned proteins are involved in intracellular membrane fusion; however, other instances of membrane fusion can also take place inter-cellularly as in myoblast fusion during skeletal muscle development. Only recently, two fusogens, myomaker and myomerger, have been identified that are indispensable for mammalian myoblast fusion. CRISPR knockout of these fusogens in mice and zebrafish leads to complete block of myoblasts fusion or embryonic lethality [82,83,84,85,86,87]. Notably, unlike the previously mentioned small fusogenic proteins, myomaker and myomerger must complement each other to complete the fusion process. For instance, myomaker mediates fusion between myoblasts or between myoblasts and fibroblasts but not fibroblasts alone [84]. However, when myomaker is co-expressed with myomerger, it is sufficient to induce fusion in non-fusogenic fibroblasts [82,85,86]. From several reports, it is evident that myomaker is needed in both fusing membranes, whereas myomerger is required only in one of the two fusing membranes [82,85]. Although the two proteins are sufficient to mediate the fusion of non-fusogenic cells, the mechanisms by which they mediate membrane destabilization or lipid mixing are yet to be elucidated.

### 5.1. Myomaker

As mentioned, myomaker is a muscle-specific membrane protein. A variety of in vivo and in vitro studies has confirmed the indispensable role of myomaker in muscle development and regeneration [83,84,88]. Myomaker is highly conserved across vertebrate organisms [84,89]. It contains a short extracellular N-terminus, seven transmembrane domains with many hydrophobic amino acids, and a C-terminus in the cytoplasm (Figure 1C). Through co-staining with various organellar markers, myomaker is found to localize mainly in the Golgi apparatus, post-Golgi vesicles, and the plasma membrane. The C-terminal cysteine residues are palmitoylated, which is important for protein trafficking through the Golgi and membrane localization. In addition, these palmitoylated C-terminal cysteine residues may also interact with C-terminal hydrophobic leucine residues for maximum protein function [90]. Moreover, the CAAX domain at the C-terminus is essential for the myomaker membrane targeting [91].

### 5.2. Myomerger

Myomerger (also known as myomixer and minion), simultaneously isolated by three independent groups, is an 84-aa fusogenic micro-peptide that is normally expressed on the cell surface (Figure 1C) [82,85,86]. Myomerger is essential for myoblast fusion because its knockout in C2C12 myoblasts caused defective pore formation [92]. Conserved in vertebrate species, myomergers are encoded by two transcripts—myomerger-S (84aa) and myomerger-L (108aa). The two isoforms encoded by both transcripts have identical fusogenic activity. Myomerger is anchored on the cell surface through a single transmembrane domain, while the N-terminus located within the cytoplasm and the long C-terminus protruding into the extracellular space. The N-terminal α-helical region may dictate the spatial orientation of myomerger in the plasma membrane, while C-terminal ectodomain plays a role in membrane destabilization [92]. Of note, the C-terminal ectodomain is predicted to be composed of two helical regions. The amphipathic helix in the first helical region could be indispensable for this activity. Despite the limited understanding of the myomerger, it is, after all, the smallest fusogen reported for cell–cell fusion.

## 6. Fusion-Associated Small Transmembrane Proteins

Unlike enveloped viruses, non-enveloped viruses do not encode fusion proteins, except for the family of reoviruses. The reovirus family encodes a small, unstructured protein known as the fusion-associated small transmembrane proteins (FAST) to mediate cell–cell fusion but not virus–cell fusion. These proteins are thought to have evolved to aid in intercellular dissemination by syncytium formation, and rupture of large syncytium helps in virus release [93]. A few examples of FAST proteins are the Avian orthoreovirus p10, Reptilian orthoreovirus p14, and Baboon orthoreovirus p15 [94]. These FAST proteins are approximately 100–200 amino acids in length and do not possess any sequence identity. However, all the proteins contain similar motifs and regions that mediate membrane fusion [95]. Each of the FAST proteins possesses an N-terminal ectodomain, a transmembrane domain, and a C-terminal endodomain (Figure 1D). Following secretion from the ER and Golgi complex, FAST proteins localize to the plasma membrane with its N-terminus extending into extracellular space and C-terminus present within the cytoplasm [95]. Unlike the SNAREs, atlastin, and spanins, FAST proteins do not interact with any receptors on the target membrane, but they use the cell’s adhesion proteins, such as cadherins, to bring the opposing membranes in close opposition [96]. In the pre-fusion and fusion stage, FAST proteins multimerize in lipid rafts along with cholesterol to mediate lipid interaction and membrane curvature [97,98,99].

### 6.1. FAST Protein Ectodomain and Membrane Proximal External Region

The N-terminal ectodomain in FAST proteins encompasses approximately 20–40 hydrophilic and hydrophobic residues and is considered to be the fusion peptide. The ectodomain along with the membrane-proximal external region (MPER) plays a role in membrane curvature and nascent pore formation [94]. FAST proteins, p10, p14, and p15, perform the same function; however, harboring remarkably diverse structures. The ectodomain of p10 contains two cysteine residues that form an intramolecular disulfide bond creating an 11-residue amphiphilic loop with conserved valine, isoleucine, and phenylalanine in the loop facilitating membrane insertion [100]. Moreover, the di-cysteine motif is palmitoylated, and that loss of palmitoylation leads to a loss of fusion activity [101]. The MPER in p10 plays a role in protein multimerization, and this multimerization in the plasma membrane is required for subsequent membrane fusion [98,102]. On the other hand, the p14 and p15 FAST proteins utilize two very different types of fusion peptides, both of which require N-terminal myristoylation and function in a sequence-specific manner to destabilize lipid bilayers [103,104,105]. Interestingly, the p14 ectodomain assumes different structures depending on the modification status. The soluble non-myristoylated p14 ectodomain peptide involves a seven-residue proline-hinged loop with conserved phenylalanine and valine residues near the tip of the loop flanked by a disordered 25-residue MPER extending in the extracellular space. However, the proline loop region of myristoylated p14 ectodomain becomes disordered and the MPER assumes a flexible amphipathic helix-kink-helix structure. Of note, the myristoylated p14 ectodomain peptide, but not a non-myristoylated version of the same peptide mediated extensive lipid mixing in the liposome fusion assay [106]. Conversely, p15 ectodomain assumes consistent structures that contain a proline-rich motif (PPAPP) that exists as a left-handed polyproline type II helix, playing a role in lipid anchoring and membrane fusion [107]. Proline residues are tolerant of substitution but multiple substitutions that disrupt the helix structure blocking p15 function.

### 6.2. FAST Protein Transmembrane Domain

Transmembrane domains (TMDs) of FAST proteins consist of 19–23 amino acids and function as reverse signal anchors to direct the localization of the proteins to the plasma membrane in an N_out_/C_in_ topology [108]. In addition to membrane anchor, the FAST protein’s TMDs drive fusion. The TMD of *reptilian reovirus* p14 is interchangeable with that of other FAST proteins but not with the TMD of heterologous proteins, such as Influenza HA or VSV-G, suggesting that FAST proteins may contain family-specific structural features in their TMDs that directly affect fusion activity [108]. On the contrary, the p15 TMD is not interchangeable with the TMD of other FAST proteins, implying that unique attributes of the p15 TMD must be maintained to mediate fusion along with its specific ecto-and endodomains [109].

### 6.3. FAST Protein Endodomain

The endodomain of FAST proteins consists of a polybasic motif (PB), an amphipathic helix (AH), and a C-terminal intrinsically disordered tail. Several research studies suggest that the endodomain interacts with cellular partners to mediate fusion. For example, the p14 endodomain interacts with Annexin A1 and in the presence of calcium ions to mediate pore expansion [95]. Additionally, p14 interacts with adaptor protein Grb2 to initiate actin polymerization, driving membrane protrusion, and fusion [110]. Studies like this may address why C-terminal truncation of p14 and p10 inhibits pore expansion and syncytium formation [111].

While the endodomain interacts with cellular partners, the PB motif of a FAST protein, such as p14, is required for Golgi transport and plasma membrane localization [112]. The PB motif could also play a more direct role in the fusion reaction through electrostatic interactions with anionic phospholipids in the cytoplasmic leaflet of membrane bilayers, as occurs during intracellular membrane fusion events [113].

AH is the most important region that promotes pore formation. For instance, the p15 endodomain AH functions as a lipid packing sensor to lower the energy barrier between two opposite membranes to form a fusion pore. Mutations that disrupt AH motif in the p15 ablate cell–cell fusion, and this motif can be functionally replaced by the predicted linear AH in the p14 endodomain, and by several cellular lipid packing sensors [114]. This study indicates that the AH motif of the FAST proteins serves to induce pore formation during membrane fusion. Indeed, among small fusogens discussed in this review, the AH exists in almost all.

## 7. Conclusions

In this review, we have summarized the core regions of a set of small-molecular-weight fusogens. Among many excellent reviews published on membrane fusion, most of them dealt with well-studied viral and intracellular fusogens; even though some focused on the specific small fusogens, (i.e., FAST proteins, myomerger) [94,115], almost no reviews were dedicated to all small fusogens. Thus, this review is timely.

Even though the mechanisms of membrane fusion have been established and well described, from the available literatures, it is easily noticed that the fusogens take advantage of related structures, such as N-terminal α-helixes for class I viral fusogens and β-sheets structure for class II viral fusogens, to promote membranes merger through stages of close apposition, hemifusion, and fusion pore formation/expansion. Clearly, diverse fusion proteins employ similar general protein structures to mediate membrane fusion, emerging the possibility that specific structures or motifs determine the fusogens’ function.

We compared the structurally compact fusogens that have evolved independently and explained the key features of each protein in Table 1. Although the aforementioned proteins do not share a structural similarity, they contain comparable motifs that have evolved to mediate homotypic or heterotypic membrane fusion in a wide variety of organisms. Some common features are seen in these proteins. Noticeably, the presence of hydrophobic residues and amphipathic helices (AH) in the fusion peptides or membrane-proximal external regions is of primary importance in the foregoing fusogens-mediated membranes fusion. The amphipathic helices characterized by the arrangement of hydrophobic and polar residues at the polar-non-polar interface makes the protein well suited for membrane binding [116,117]. In addition, AHs are able to sense membrane curvature, which has been believed to be an important modulator of the fusion process [118,119]. Thus, it is not surprising that the amphipathic helix has been found in many fusogens. Furthermore, mutations of hydrophobic residues within AHs to hydrophilic residues inhibited the abilities of AHs inducing membrane curvature and binding to lipid membranes, while mutations of hydrophobic residues to more bulky hydrophobic residues promoted such functions [120]. Considering the ubiquity of AH in the compact-structural fusogens and its unique properties, here we suggest, for the first time to our knowledge, that the amphipathic helix may act as a characteristic structure of fusion proteins. The overall structure—rather than the primary sequence—is conserved based on research studies on well-studied viral fusion proteins. Thus, we believe it is important to use structural information to search for new fusion proteins. However, the identification of fusogens is challenging and requires a complex analysis depending on diverse experimental approaches to evaluate fusogenic activities. Even though a scoring system was suggested by the other reviews based on the fusogenic activities [7], here we proposed a new point on the prediction of fusogens. In conclusion, we hope that this review may facilitate the discovery of new membrane fusion proteins and provide new insights into the mechanisms of protein-mediated membrane fusion.

## Figures and Tables

**Table 1:** Key characteristics of small fusogens required to mediate membrane fusion.

**Fusogen**	**Autonomous**	**TMD**	**Multimer**	**Topology**	**Distribution**	**Fusion Complex**	**AH**	**HH**	**Other Motifs**	**FP**
SNAREs:	No		Heteromultimer	N_out_/C_in_	bilateral	Heterotypic	N-terminal		PB	N-terminal (both terminal for SNAP-25)
Syntaxin		1								
SNAP-25		-								
VAMP		1								
ATL	Yes	2	Homodimer	N_out_/C_out_	bilateral	Homotypic	C-terminal	N-terminal		N-terminal
i-Spanin, Rz	No	1	Homodimer	N_in_/C_out_	bilateral	Heterotypic	-	C-terminal		C-terminal
o-Spanin, Rz1	No	-	Homodimer	N_in_/C_out_	bilateral	Heterotypic	-	-	polyproline helix	
u-Spanin	Yes	1	Homomultimer	N_out_/C_in_	unilateral	Homotypic	-	-	β-barrels	
Myomaker	No	7	Unknown	N_out_/C_in_	bilateral	Heterotypic	-	-		C-terminal
Myomerger	No	1	Unknown	N_in_/C_out_	unilateral	Heterotypic	C-terminal	C-terminal		
FAST	Yes	1	Homomultimer	N_out_/C_in_	unilateral	Homotypic	C-terminal	N-terminal	PB	N-terminal

TMD: Transmembrane domain; AH: Amphipathic helix; PB: Poly-basic motif; FP: Fusion peptide; HH: Hydrophobic helix.
